# Novel bioinformatics quality control metric for next-generation sequencing experiments in the clinical context

**DOI:** 10.1093/nar/gkz775

**Published:** 2019-09-12

**Authors:** Maxim Ivanov, Mikhail Ivanov, Artem Kasianov, Ekaterina Rozhavskaya, Sergey Musienko, Ancha Baranova, Vladislav Mileyko

**Affiliations:** 1 Department of Biological and Medical Physics, Moscow Institute of Physics and Technology, Dolgoprudny 141700, Russian Federation; 2 Vavilov Institute of General Genetics, Moscow, Russian Federation; 3 Atlas Oncology Diagnostics, Ltd, Moscow, Russian Federation; 4 Research Centre for Medical Genetics, Moscow, Russian Federation; 5 School of Systems Biology, George Mason University, Fairfax, VA 22030, USA

## Abstract

As the use of next-generation sequencing (NGS) for the Mendelian diseases diagnosis is expanding, the performance of this method has to be improved in order to achieve higher quality. Typically, performance measures are considered to be designed in the context of each application and, therefore, account for a spectrum of clinically relevant variants. We present EphaGen, a new computational methodology for bioinformatics quality control (QC). Given a single NGS dataset in BAM format and a pre-compiled VCF-file of targeted clinically relevant variants it associates this dataset with a single arbiter parameter. Intrinsically, EphaGen estimates the probability to miss any variant from the defined spectrum within a particular NGS dataset. Such performance measure virtually resembles the diagnostic sensitivity of given NGS dataset. Here we present case studies of the use of EphaGen in context of BRCA1/2 and CFTR sequencing in a series of 14 runs across 43 blood samples and 504 publically available NGS datasets. EphaGen is superior to conventional bioinformatics metrics such as coverage depth and coverage uniformity. We recommend using this software as a QC step in NGS studies in the clinical context. Availability: https://github.com/m4merg/EphaGen or https://hub.docker.com/r/m4merg/ephagen.

## INTRODUCTION

Next-generation sequencing has transformed the landscape of the whole field of medical genetics. It enhanced the performance of the genetic testing as well as expanded and facilitated understanding of clinical genetics (1–4). The principal focus of the clinical geneticists remains on Mendelian diseases as they are the most well described and straightforward to integrate into clinical practice. However, decades of research efforts and routine testing shed light on the spectrum of variations in human genes, associated with a wide range of genetic disorders and their clinical significance in terms of variable penetrance and expressivity (5). Moreover, for the most wide-spread genetic diseases, numeric research collaborations and public databases provided information on common and population specific minor allele frequencies for clinically significant variants. For instance, as of May 2018, Breast Cancer Information Core database (6) contains information on relative clinically relevant variants across BRCA1 and BRCA2 genes, implicated in hereditary breast cancer development, based on the 11 344 affected population size.

Against this background, despite excessive informational yield that NGS sequencing data provides, in routine clinical practice, in essence, it is used to confirm the findings for the patient: it is either known pathogenic variants (positive result) or a wild-type (negative result). Meanwhile, the detection of a variant which is not annotated in public databases and in literature may be considered as an exceptional event. Nevertheless, such *de novo* variants, as well as variant of uncertain significance or any findings beyond the known spectrum of pathogenic variants, have limited significance for clinical practice. Taking this into account, commonly used NGS quality control metrics, such as read coverage depth or coverage uniformity, fail to elucidate the worth of the negative result since target sequencing regions may be intrinsically unequal depending on their genome position.

Here, we describe a novel approach to the measurement of performance in routine clinical NGS testing. Given single NGS dataset in BAM format and spectrum of the variants of interest with known allele prior probabilities, it employs methodology which essentially resembles variant calling under reversed null hypothesis. Instead of detecting variants, this algorithm is utilized to estimate the probability to miss any variant from the defined spectrum and, therefore, to decide whether collected data are suitable for clinical interpretation or no. Performance of presented sensitivity calculation was extensively tested both on simulated and on real-life sequencing datasets. Since it matches every dataset with a single number, it is ideally suited for routine NGS quality control and allow head-to-head comparison of different sequencing approaches.

## MATERIALS AND METHODS

### Sample collection and sequencing

Sequencing was performed on 43 blood samples from 43 breast cancer patients referred for BRCA1/2 sequencing as a part of routine case management. Participation in this project was based on informed consent. All further analyses were based on the archival data that were stored in the database with no current connection to the patients’ identifiers. The research was approved by the local ethics committee of the Atlas Medical Center, LLC. The project was conducted in accordance with the principles expressed in the Declaration of Helsinki.

Twenty milliliters of peripheral blood was collected from each patient. Circulating plasma DNA was extracted from 20 ml of plasma using Plasma DNA extraction kit (Biosilica) or QIAamp Circulating Nucleic Acid Kit (Qiagen) according to the manufacturer's protocols. DNA quality and quantity were evaluated with Agilent Bioanalyzer 2100 using High Sensetivity DNA kit (Agilent Technologies).

Target regions amplification was performed employing the Atlas ABC panel. Primer panel was designed via Ion Ampliseq Designer (Thermo Fisher Scientific Inc) through White Glove process and include two primer pools, comprising 409 amplicons within 4 cancer-related genes: BRCA1, BRCA2, ATM, CHEK2. Pooled libraries were sequenced using Ion Torrent PGM (Thermo Fisher). Raw sequence data analysis, including base calling and demultiplexing, were performed using the Torrent Suite Software v.4.0.2 (Life Technologies).

Ten nanograms of circulating plasma DNA were used to generate sequencing libraries using the Ion Ampliseq library preparation kit v2.0 (Life Technologies) according to the manufacturer's protocol. The barcoded libraries were quantified using an Agilent 2100 Bioanalyser and Qubit 2.0 Fluorometer TM (Life Technologies) and then diluted to a final concentration of 10 pM for template preparation using the OneTouch 2 instrument and Ion One Touch Template kit v2 (Life Technologies). The resulting pooled libraries were quality control checked using the Ion Sphere quality control Kit on the Qubit 3.0 Fluorometer. Libraries were sequenced on the PGM Ion Torrent (Life Technologies) using a PGM 200 sequencing kit v2 and 318 Chip v2.

All samples were sequenced within 14 sequencing runs (Figure 9). Sequence data mapping to reference genome (GRCh37.p13) was performed with Burrows-Wheeler Aligner (BWA-mem, version 0.7.7-r441) (7).

### Statistical framework

At first, we consider that object of testing is known to harbor pathogenic variants of particular, limited spectrum. This consideration is based on the methodology used to judge whether variant is clinically relevant or no, requiring population data, segregation data, functional data and so on (8). Assuming that this spectrum of pathogenic variants is generated based on the previously studied affected population ${P_{aff}}$ of size ${N_{{P_{aff}}}}$ we can define a set of *M* mutation sites with known allele count at each site *m*, ${n_m}:\ \mathop \sum \nolimits_{m\ = \ 1}^M {n_m} = {N_{{P_{aff}}}}$. Therefore, relative allele frequency at site m is ${p_m} = {n_m}\ /{N_{{P_{aff}}}}$. Given single sequencing dataset ${\boldsymbol{D}}$, covering aforementioned *M* sites we can define the probability to miss any variant *m*. This estimation resembles quasi-experiment to detect mutations in population ${P_{aff}}$, while the average dataset quality during this experiment is the same as the quality of the dataset ${\boldsymbol{D}}$. Here, dataset quality is defined by read count covering each position *m* and base quality. In this context, we can define *in silico* sensitivity of the dataset, resembling diagnostic sensitivity.

EphaGen takes aligned sequencing data in .bam format as input file as well as pathogenic variant spectrum in .vcf format, describing *M* mutation sites with the allele count ${n_m}$ for each. To estimate probability to miss variant *m* we employ simple probabilistic model for variant calling (9). Given sequencing data may be represented by matrix ${\boldsymbol{D}}\ = \ {( {{{\bar{D}}_1}, \ldots ,{{\bar{D}}_M}} )^T}$, totaling *N* reads covering *M* target mutation sites, with $\overline {D_m} \ = \left( {\underbrace {1,,,1}_{l_m}},{\underbrace {0, \ldots ,0}_{k_m- l_m}}\right)$, representing alignment for the site m, where 1 stands for reference allele and 0 stands for alternative allele. Assuming that (i) data $\overline {{D_m}}$ at different sites are independent (ii) sequencing errors arise independently at rate ${\rm{\varepsilon }}$ and (iii) error rates are identical for all bases, posterior probabilities for observing data $\overline {{D_m}}$ at site *m* given 0, 1 or 2 reference alleles at this site can be approximated with a binomial distribution:

(1) $$\begin{equation*}P\ \left( {{\boldsymbol{D}}{\rm{|}}{0_m}} \right) = \ \left( {\begin{array}{@{}*{1}{c}@{}} {{k_m}}\\ {{l_m}} \end{array}} \right){{\rm{\varepsilon }}^{{l_m}}}\ {\left( {1 - {\rm{\varepsilon }}} \right)^{{k_m} - {l_m}}}\end{equation*}$$

(2) $$\begin{equation*}P\ \left( {{\boldsymbol{D}}{\rm{|}}{1_m}} \right) = \ \left( {\begin{array}{@{}*{1}{c}@{}} {{k_m}}\\ {{l_m}} \end{array}} \right)/{2^{{k_m}}}\end{equation*}$$

(3) $$\begin{equation*}P\ \left( {{\boldsymbol{D}}{\rm{|}}{2_m}} \right) = \left( {\begin{array}{@{}*{1}{c}@{}} {{k_m}}\\ {{l_m}} \end{array}} \right)\ {\left( {1 - {\rm{\varepsilon }}} \right)^{{l_m}}}\ {{\rm{\varepsilon }}^{{k_m} - {l_m}}}\end{equation*}$$

For the posterior probability of observing 0, 1 or 2 reference alleles at site *m* given data $\overline {{D_m}}$ we have:

(4) $$\begin{equation*}P\ \left( {{\alpha _m}{\rm{|}}{\boldsymbol{D}}} \right) = \frac{{P\left( {{\boldsymbol{D}}{\rm{|}}{\alpha _m}} \right)P\left( {{\alpha _m}} \right)}}{{\mathop \sum \nolimits_{{i_m} = 0}^2 P({\boldsymbol{D}}|{i_m})P\left( {{i_m}} \right)}}\ ,\ \alpha \in \left\{ {0,1,2} \right\}\end{equation*}$$

And called genotype is thus:

(5) $$\begin{equation*}\widehat {{\alpha _m}} = argma{x_{{\alpha _m} \in \left\{ {0,1,2} \right\}}}\ P\left( {{\alpha _m}{\rm{|}}{\boldsymbol{D}}} \right),\end{equation*}$$

While mutant genotype $\widehat {{\alpha _m}} = \ 0$ or $\widehat {{\alpha _m}} = \ 1$ are accepted if $P( {\widehat {{\alpha _m}}{\rm{|}}{\boldsymbol{D}}} ) >\ P( {\widehat {{\alpha _m}}} )$ and genotype Phred quality exceeds 20:

(6) $$\begin{equation*}{Q_{\widehat {{\alpha _m}}}} = \ - 10\ lo{g_{10}}\left[ {1 - \overline {P\left( {\widehat {{\alpha _m}}{\rm{|}}{\boldsymbol{D}}} \right)} } \right] >20,\end{equation*}$$

where $\overline {P({{\hat{\alpha }}_m}| {{\boldsymbol{D}})} } \, = \,\frac{{P({{\hat{\alpha }}_m}| {{\boldsymbol{D}}) - } P({{\hat{\alpha }}_m})}}{{(1 - P({{\hat{\alpha }}_m}))}}$

Prior probabilities of homozygous for alternative allele call, heterozygous call and homozygous for reference allele call can be calculated under Hardy-Weinberg equilibrium and are $P\ ( {{0_m}} ) = {p_m}^2\ ,\ P\ ( {{1_m}} ) = \mathop \sum \nolimits_{i = 1,i \ne m}^M 2\ {p_i}\ {p_m}\ = \ 2\ {p_m}( {1 - {p_m}} )$ and $P\ ( {{2_m}} ) = \mathop \sum \nolimits_{i = 1,i \ne m}^M {p_i}^2\ + \ \mathop \sum \nolimits_{i\ = \ 1,i \ne m}^M \mathop \sum \nolimits_{j\ = \ 1,j \ne i}^M 2\ {p_i}\ {p_j} = {( {1 - {p_m}} )^2}\ {\rm{respectively}}.$

According to the aforementioned quasi-experiment, each site is tested to be mutant (homozygous or heterozygous for alternative allele) or reference (homozygous for reference allele). Considering that true condition is presence of alternative allele in homozygote or heterozygote, sensitivity can be calculated as ratio of number of true positives (quasi-test system calls mutation if allele is present in sample) to the total count of alleles:

(7) $$\begin{equation*}S\ = \frac{1}{2}\ \mathop \sum \nolimits_{m\ = \ 1}^M \left[ {{\gamma _{m,1}}P\left( {{1_m}} \right) + 2*{\gamma _{m,0}}\ P\left( {{0_m}} \right)} \right],\end{equation*}$$

where ${\gamma _{m,1}}\ {\rm{and}}\ {\gamma _{m,0}}$ are probabilities to correctly identify sample as heterozygous and homozygous for alternative allele at site *m* respectively.

During aforementioned quasi-experiment we may expect any data ${{\boldsymbol{D}}_\varphi }\ = \ {( {{{\bar{D}}_{1,\varphi }}, \ldots ,{{\bar{D}}_{M,\varphi }}} )^T}$, where probabilities of observing each data $\overline {{D_{m,\varphi }}}$ may be calculated by equations 1–3. For each possible dataset ${{\boldsymbol{D}}_\varphi }$ using equations 5, 6 we can estimate whether the variant *m* will be detectable or no. Therefore, accounting for all possible datasets that may be generated, probability to correctly detect $\alpha$ ($\alpha \in \{ {0,1} \}$) alternative alleles for the site *m* may be calculated as ${\gamma _{m,\alpha }} = \mathop \sum \nolimits_\varphi P( {\overline {{D_{m,\varphi }}} {\rm{|}}{\alpha _m}} )\ {\delta _{\varphi m,\alpha }}P( {{\alpha _m}} )$,

where, ${\delta _{\varphi m,\alpha }}$ denotes where variant *m* is detectable in dataset ${{\boldsymbol{D}}_\varphi }$ or no:

$$\begin{equation*}\ {\delta _{\varphi m,\alpha }} = \ \left\{ {\begin{array}{@{}*{1}{c}@{}} {1,if\ \alpha \ = \widehat {{\alpha _m}}\ }\\ {0,if\ \alpha \ne \widehat {{\alpha _m}}} \end{array}} \right.,\ \alpha \in \left\{ {0,1} \right\}\end{equation*}$$

And, thus, Equation (7) can be written as:

(8) $$\begin{eqnarray*}S &=& \frac{1}{2}\ \mathop \sum \nolimits_{m\ = \ 1}^M \mathop \sum \nolimits_\varphi \big[ {P\left( {\overline {D_{m,\varphi}} {\rm{|}1_m}} \right)}\ {\delta_{\varphi m,1}}P({1_m})\nonumber\\ &&+ 2*P\left( {\overline {{D_{m,\varphi }}} {\rm{|}}{0_m}} \right)\ {\delta _{\varphi m,0}}\ P\left({0_m} \right) \big] = \mathop \sum \nolimits_{m = 1}^M \mathop \sum \nolimits_\varphi {c_{m,\varphi }}\nonumber\\ \end{eqnarray*}$$

In Equation (8), the second sum is over all combinations of data $\overline {D_{m,\varphi}}$ assuming $i$ alternative alleles. Assuming that all reads are equal and discounting differences in base quality it can be approximated with:

(9) $$\begin{equation*}S\ = \mathop \sum \nolimits_{m = 1}^M \mathop \sum \nolimits_{i = 0}^{{k_{m,\varphi }}} {c_{m,\varphi }}\ ,\end{equation*}$$

Where $\overline {D_{m,\varphi}} = \overline {D_{m,i}} \equiv \left({\underbrace {1,,,1}_i},{\underbrace {0, \ldots ,0}_{k_{m,\varphi}-i}} \right).$

Further, we use a Poisson distribution with cumulative distribution function $cdf ( {{k_{m,e}}} ) = \ \frac{{\Gamma(\lfloor {{k_{m,e}}\rfloor,{k_m}} )}}{{\Gamma( {{k_m}} )}},\ {k_{m,e}} >0$ as a model to calculate probability of observing ${k_{m,e}}$ reads covering site *m* after generating another dataset ${{\boldsymbol{D}}_\varphi }$ with the same total amount of *N* reads covering *M* target mutation sites. To define the set of expected coverages at site *m*, ${K_{m,exp}}$, we can calculate the pair of ${k_{m,l}}$ and ${k_{m,h}}$ so that ${K_{m,exp}} = \ \{ {k:{k_{m,l}} \le k \le {k_{m,h}}} \}$, ${k_{m,l}} - {k_{m,h}}$ is minimal across all possible pairs and:

$$\begin{eqnarray*}\Big\lbrace \begin{array} {cc}CDF\left( {{k_{m,h}}} \right) - CDF\left( {{k_{m,l}}} \right) \ge 0,99&\\[-6pt] &\!\!,\,{k_{m,h}} >{k_m},{k_{m,l}} > 0, {\rm{or}}\\[-6pt] {{k_{m,h}} - \ {k_m} = {k_m}\ - {k_{m,l}}}\end{array}\\ CDF\left( {{k_{m,h}}} \right) - CDF\left( {{k_{m,l}}} \right) \ge 0,95,\,{k_{m,h}} > {k_m},\,{k_{m,l}}\, = \,0,\end{eqnarray*}$$

which stands for the 99% confidence interval of ${k_{m,e}}$. After ${K_{m,exp}}$ defined this way, probability of observing ${k_{m,e}} \in {K_{m,exp}}$ should be normalized according to the chosen interval: $P\ ( {{k_{m,e}}} ) = \ pmf( {{k_{m,e}},\ {k_m}} )/\mathop \sum \limits_{k \in {K_{m,exp}}} pmf( {k,\ {k_m}} )$, where $pmf( {{k_{m,e}},\ {k_m}} )$ is probability mass function of Poisson distribution.

Here, we should note, that coverage for mutation site may equals to 0. Since it may be caused by amplicon drop-out, deeper sequencing of the same library wont naturally generate data with reads covering this site. On the other hand it may be caused by significant coverage non-uniformity or low mean coverage and thus, despite not observed in data ${\boldsymbol{D}}$, it might be covered during sequence generating $\tilde{N} >N$ reads, though based on the single sequencing dataset $\tilde{N}$ remains not-definable. Therefore, for those sites, covered with 0 reads it is defined that ${k_{m,l}} = \ \ {k_{m,h}} = \ 0$ and ${K_{m,exp}} = \ \{ 0 \}$ (10).

After defining ${K_{m,exp}}$ we can update Equation (6):

$$\begin{equation*}S\ = \mathop \sum \limits_{m = 1}^M \mathop \sum \limits_{{k_{m,e}} \in {K_{m,exp}}} \mathop \sum \limits_{i = 0}^{{k_{m,e}}} {c_{m,\varphi }}\ P\left( {{k_{m,e}}} \right)\ \end{equation*}$$

It is known that the process of sequence analysis is prone to produce diverse systemic errors originating at different stages of analysis, including directly sequencing, library preparation and even specimen sampling. Such errors can be handled employing more complicated variant calling models, cross-sample analysis or other methods. However simple probabilistic model may produce false calls in such cases, which will introduce bias into sensitivity calculation. In order to avoid such perturbation, during the step of calculating ${k_m}$ for each site if homozygote or heterozygote for alternative allele is more likely than homozygote for reference allele, all reads supporting alternative allele are purged from the alignment.

## RESULTS

### Overview

**Figure 1.**
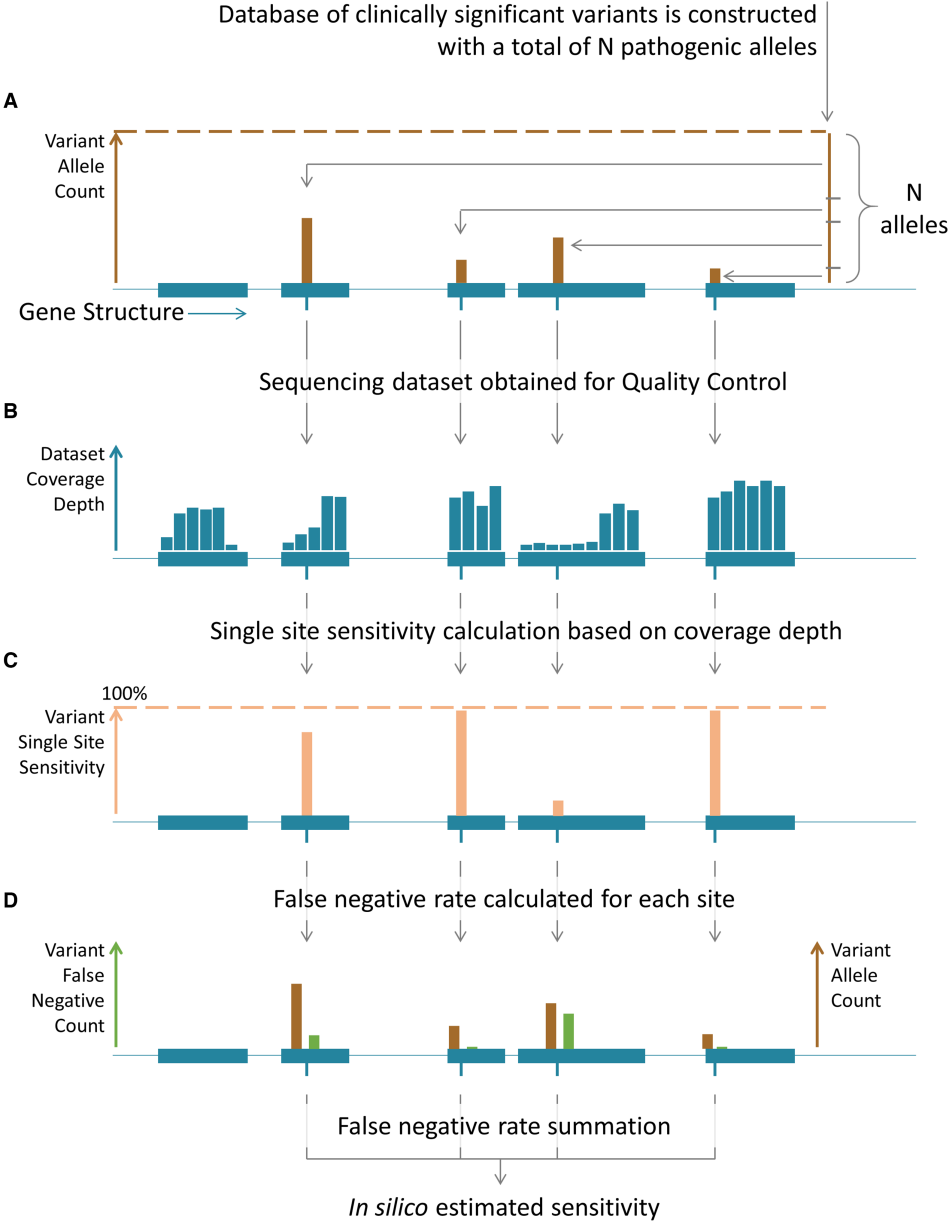
Overview of the EphaGen framework for calculation of the estimated in silico sensitivity. (**A**) Reference database of target clinically significant variants is constructed with information on the allele count for each present allele at every variant site. (**B**, **C**) Based on the simple probabilistic variant calling model taking into account site coverage, base quality and allele prior probability the probability to miscall each implied variant allele from reference database are calculated, contributing to the Single Site Sensitivity or false negative rate related to the single variant allele subtracted from 1. (**D**) Finally, resulting dataset sensitivity is calculated through summation across all sites (see Methods).

We have developed EphaGen, an open-source application implemented in Perl/R, which can be used as a stand-alone version. EphaGen takes two files as input: aligned sequenced data in .bam format and target variant spectrum in .vcf format. Target variant spectrum can be constructed based on the previously studied affected population employing prospective, retrospective, meta-analysis or database view methodology. Therefore, each variant observed in the affected population can be associated with minor allele count which should be included into input .vcf file. Based on the simple probabilistic model for variant calling under the reversed null hypothesis, EphaGen estimates the probability to miss any variant from target spectrum or single site sensitivity (Figure 1). Therefore, instead of directly detecting variant from sequencing data, EphaGen calculates maximum potential diagnostic sensitivity of any variant calling methodology which can be employed to detect variants from data (see Methods for detailed statistical framework).

**Figure 2.**
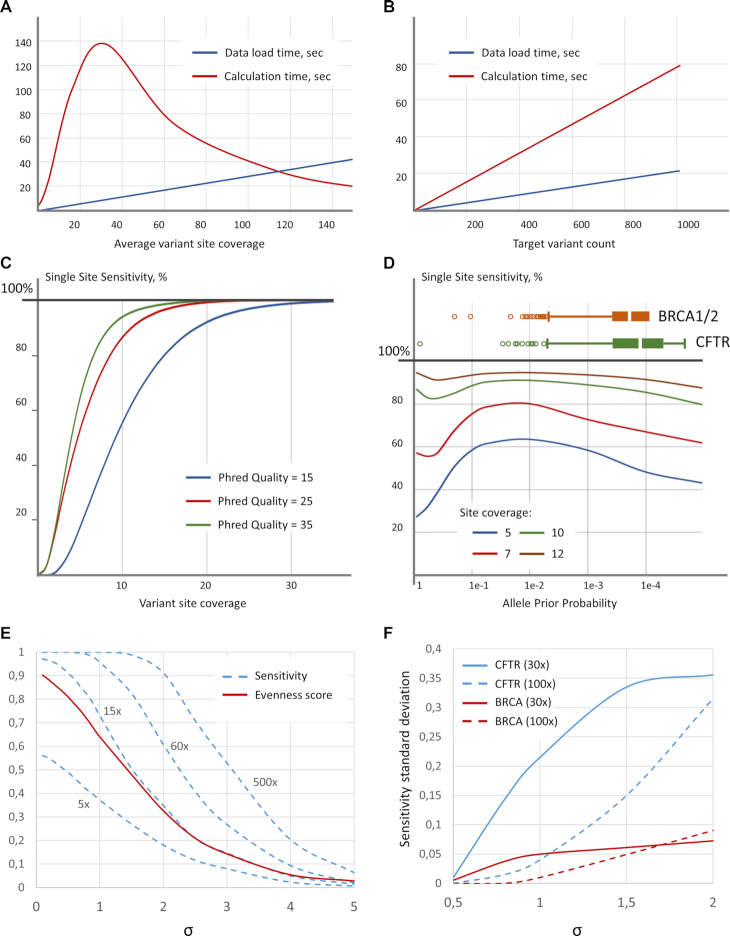
EphaGen validation on simulated data. (A, B) EphaGen run time is limited to two time-limiting stages: dataset load and directly sensitivity calculation based on the statistical framework. Dependency of both of them on average variant site coverage is built for 1000 target variants (**A**) and on target variant count is built for 100× average variant site coverage (**B**). (**C**) Single site sensitivity calculation varying variant site coverage demonstrates S-shape curve with increasing steep as the phred quality score increases. Graph is built at allele prior probability of 0.015%. (**D**) Single site sensitivity calculation varying allele prior probability demonstrates a peculiar curve however distinguishing an increase in sensitivity as the allele prior probability increases at range 0–5% with a decreasing slope as the coverage increases. Histograms at the top of the graph demonstrates allele prior probability distribution for reference databases of target clinically significant variants build based on BIC and CFTR2 databases for BRCA1/2 and CFTR genes respectively. Influence on coverage uniformity was assessed by simulating data with log-normal coverage distribution across fixed count of variant sites with varying standard deviation (σ) and fixed mean coverage at 5×, 15×, 60× and 500×. Variant allele prior probabilities followed uniform distribution during simulation. (**E**). Simulating data with allele prior probability distribution resembling the ones for BRCA1/2 and CFTR variants introduces variation in sensitivity (**F**), which increases as coverage distribution standard deviation grows up.

EphaGen run time depends on both the amount of reference variant sites and read count generated during NGS experiment (Figure 2A, B). While dataset load and sensitivity calculation based on the statistical framework are two time-limiting stages of the analysis both of them pertain linear growth depending on the target variant sites count. Calculation time follows exponentials growth as average reference variant site coverage increases. To eliminate drastic escalation of the calculation we used empirically derived single site sensitivity approximation based on the generalized logistic curve.

### Performance of sensitivity estimation on simulated data

The crucial component of EphaGen is a pre-compiled VCF-file database of genomic variant alleles with the corresponding probabilities to identify each variant allele in the affected population. In our study, we used BIC (Breast Cancer Information Core, assessed may 2018) (6) and CFTR2 (Clinical and Functional Translation of CFTR, assessed may 2018) (11) databases to compile two target variant spectrums in order to test efficiency of diverse NGS-based systems to detect BRCA1/2 and CFTR clinically significant variants respectively. Only variants noted as clinically important in BIC and CF-causing or those with varying clinical consequence in CFTR2 were used for the analysis excluding variants with the reference or alternative allele of 50 bp or longer. A total of 1319 various variant sites were collected for BRCA1/2 genes amounting to 1339 diverse alleles comprising 11 344 alleles counts and 310 variant sites for CFTR gene (321 diverse alleles and 136 260 alleles counts).

Single site sensitivity calculation is based only on three variables: site coverage, base quality and allele prior probability, calculated as the site allele count divided by the total allele count in reference VCF-file (see Methods for details). Therefore, we set to evaluate the performance of this statistical framework varying the aforementioned parameters. The dependency of single site sensitivity on the site coverage built at 0.015% allele prior probability follows classic S-shape curve reaching 99% sensitivity at 41×, 26× and 21× site coverage at 15, 25 and 35 base Phred quality respectively (Figure 2C). Meanwhile, dependency on the allele prior probability follows peculiar form, demonstrating the growth of the sensitivity as allele probability increase in a range 0–5% comprising the major fraction of allele probability distribution across diverse genes with the heterogenic clinically significant variant spectrum (Figure 2D).

In order to estimate the dependency of the sensitivity calculation on coverage uniformity, we simulated coverage following log-normal distribution with varying mean and standard deviation (σ) for the set of 100 variant sites (Figure 2E). Allele prior probabilities were simulated with the uniform distribution. Base Phred quality was constant and set to 30. To measure coverage uniformity, we used evenness score, essentially described in Oexle (12). Evenness score calculation was performed across all reference variant sites. At low coverage depth, sensitivity retains almost constant ration to evenness score when standard deviation ranges from 0.1 to 1, which is the most common range for NGS coverage uniformity. However, at high coverage, as expected, sensitivity asymptotically approaches 100%, meaning that despite decreasing coverage uniformity all sites are covered with enough reads to confidently detect every single variant.

Further instead of uniform distribution for variant prior allele probability we use the distribution that follows previously collected BRCA1/2 of CFTR sites (Figure 2D). This resulted in variation introduced into sensitivity calculation. As coverage uniformity decreases, standard deviation of sensitivity across different data simulations demonstrates growth, while the rate depends on the coverage depth (Figure 2F). In addition, sensitivity variation depends on the spectrum of variant allele frequencies. For instance, the standard deviation of the sensitivity to detect CFTR variants across different data simulation is 3.6 times higher than for BRCA variants, which is in agreement with the difference between allele frequency scatter for two reference databases (coefficient of allele count variation: 12.8 versus 8.3 for CFTR and BRCA respectively). This means that the more allele frequency distribution resembles uniform distribution, the less variation of sensitivity across datasets is observed.

### The utility of estimated sensitivity as NGS performance measure

**Figure 3.**
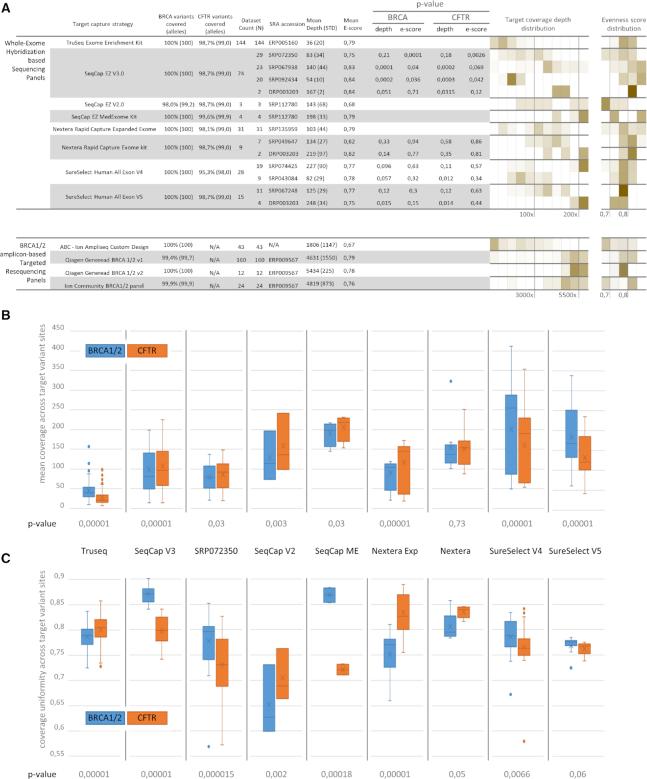
Data used for EphaGen validation. (**A**) A total of 504 publically available datasets from 11 different SRA projects were downloaded to estimate sensitivity calculation performance, representing 11 different target capture strategies. In addition, 43 datasets were generated employing Ampliseq Custom Design panel, targeting only BRCA1/2, ATM and CHEK2 genes. Diverse panels were characterized with diverse coverage of reference variant database (see main text for calculation methods), thus, limiting their sensitivity for detection of thereof. *P*-values calculation was based on t-test for difference between parameter distribution across datasets from single project versus parameter distribution across datasets generated employing specific panel, excluding datasets from that project. Histograms (**B**, **C**) demonstrates differences between parameter distribution (mean coverage – B and coverage uniformity calculated as evenness score – C) across different loci, noting that coverage uniformity is far from ubiquitous across the whole target regions.

In order to assess the performance of the EphaGen statistical framework, we have downloaded NGS sequencing data from 11 SRA studies (Figure 3A) with a total of 308 whole exome sequencing datasets and 196 target sequencing datasets. This comprised eight different whole exome sequencing (WXS) panels and three BRCA1/2 targeted panels. In addition to publically available datasets, we have performed 14 sequencing runs for target resequencing across a total of unselected 43 blood samples by leveraging custom amplicon panel designed to cover all exons of BRCA1 and BRCA2. As all analyzed publically available datasets were obtained employing Illumina sequencing technology, generating generally the same base quality, calculated *in silico* sensitivity was related to two major bioinformatics quality control metrics: mean target coverage depth and coverage uniformity defined by evenness score.

Considering that WXS datasets were collected across different laboratories, average coverage depth significantly varied across different datasets. However, that does not relate to evenness scores which were generally similar across different datasets for particular capture panel, with an exception of SRP072350 project utilizing SeqCap V3.0 (mean evenness score of 0.75 versus 0.84). This indicates that library preparation strategy has a potential to significantly impact resulting sequencing efficiency of specific loci. Despite similar GC content between target regions mapping to BRCA1/2 and CFTR loci, most panels demonstrated slight differences of mean loci coverage depth (mean ratio 1.24 with the maximum at 1.7 for TruSeq Exome Enrichment Kit) (Figure 3B). Importantly, coverage of different loci across most panels demonstrated significantly different uniformity with mean evenness score difference of 0.05 and reaching 0.07 and 0.08 for SeqCap EZ V3.0 and Nextera Rapid Capture Expanded Exome kits respectively (Figure 3C), illustrating the need to thoroughly control coverage uniformity across all target loci during multi-genic analysis rather than focusing on genome-wide coverage uniformity.

Although the panels selected for the analysis pertain whole exome design or BRCA1/2 targeted design, some of the variants from the pre-compiled variant databases may be not targeted by any of the panel. This may result in inconsistent coverage of such variant sites generated by these panels and therefore cap the sensitivity calculation for some datasets. Hence, we sought to estimate such sensitivity cap for each panel for both BRCA1/2 and CFTR variants. For that, we counted sites covered by at least 5 or 0.1× (mean locus coverage depth) reads across at least 50% downloaded datasets for each panel. As a result, every panel covers 100% of BRCA1/2 alleles with an exception for SeqCap EZ v2.0. Only three datasets employing indicated panel were used for the analysis. Interim, most panels reach sensitivity cap for detecting CFTR reference variants at 99% or less, mostly due to commonly mistargeting intronic variants, namely rs397508266, rs397508261 and rs75039782, comprising nearly 0.93% of all CFTR alleles (Figure 3A).

The dependency of the NGS dataset sensitivity on the mean target loci coverage may be approximated with Single Site Sensitivity curve (Figure 2C) with R2 of 0.98 and 0.52 for BRCA1/2 and CFTR variants, respectively. It is important to note that BRCA1/2 approximation reaches maximum coefficient of determination for Single Site Sensitivity curve built with Phred quality of 14, while for CFTR – 19. Nevertheless, approximation accuracy for the CFTR sensitivity calculation is low (Figure 2), with higher coefficient of variation for sensitivity at low coverage as compared to BRCA1/2 related approximation (0.22 versus 0.02, 0.13 versus 0.04 and 0.006 versus 0.03 for the coverage depth bands 10–20, 20–30 and 30–40 bp respectively). Such high sensitivity variation for CFTR datasets is in accordance with that in the simulated data (Figure 2F). However, sensitivity to detect CFTR variants reaches 99.7% of the estimated sensitivity cap at 30x-40x in average, while the same level of accuracy for BRCA1/2 variants analysis can be reached only at 50x-60x coverage (99.3% of cap in average). This could be explained by the wider spectrum of BRCA1/2 mutations with 1230 (275 for CFTR) unique variant sites spread on 24.5 kb (7.1 kb for CFTR) target region and 12.3 bp (15.0 for CFTR) average inter-variant distance within the single exon. Thus, either higher uniformity is required to confidently detect the whole spectrum or higher coverage depth with low uniformity. Meanwhile, 48% of BRCA1/2 alleles were presented with the minimal allele count of 1 in reference VCF-file database and thus missing the single variant does not lead to overall sensitivity perturbation and therewith demonstrating higher approximation accuracy. Meanwhile, each CFTR reference variant is presented with higher allele prior probability in average and thus missing it disturb overall sensitivity at a higher order compared to the BRCA1/2 case. Overall this points that different test systems demonstrate the varying response to sequencing coverage depth. Thus, reaching the same quality standard may require higher coverage depth for some test systems.

**Figure 4.**
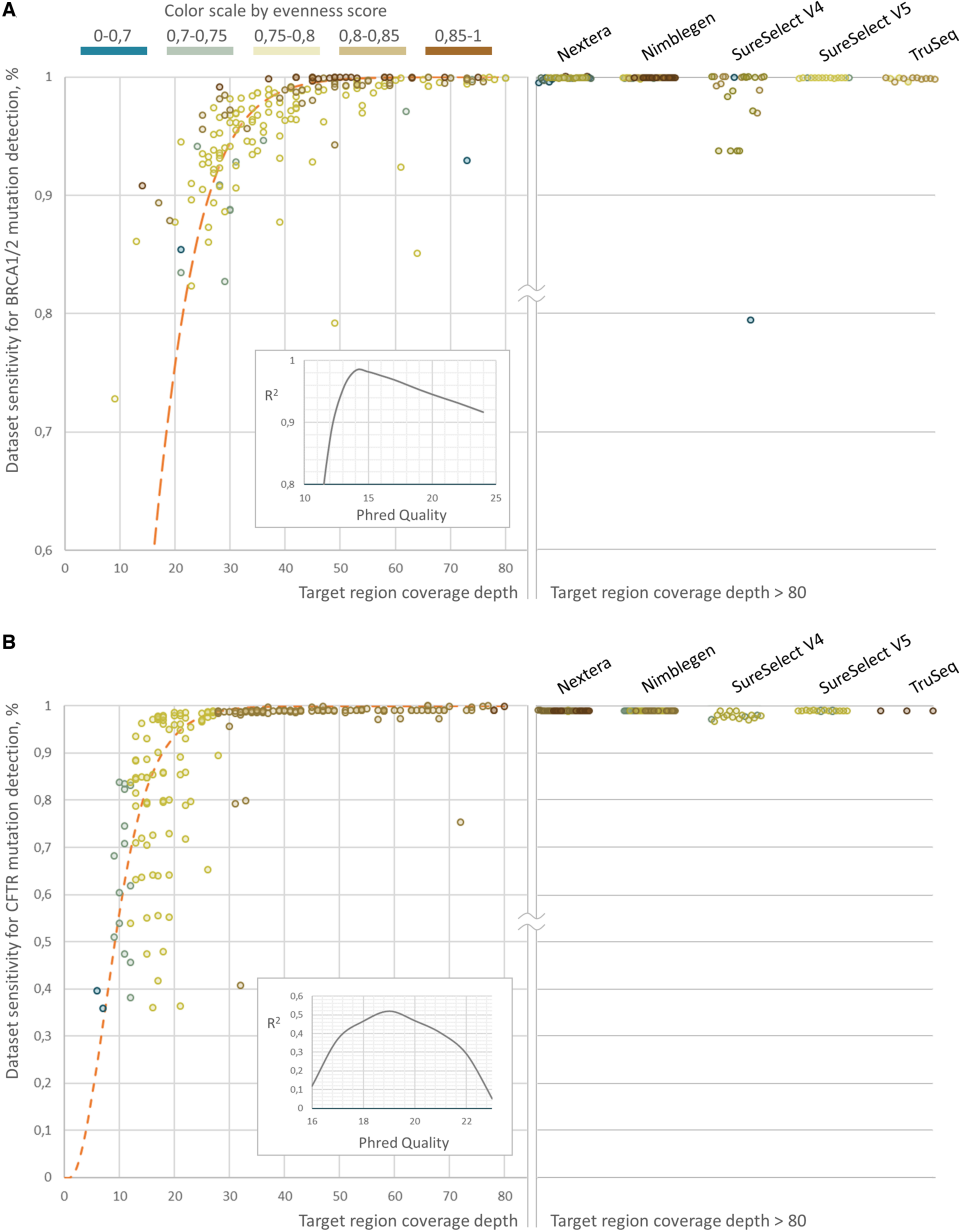
Sensitivity calculation results across all test datasets for BRCA1/2 (A) and CFTR (B) loci. Approximation was performed with the curve, resembling dependency of the Single Site Sensitivity on coverage (Figure 2B) with varying Phred Quality. Inline graphs demonstrate approximation accuracy at different Phred Quality values.

Further datasets demonstrating outlying *in silico* sensitivity were identified by employing Chauvenet's criterion under the assumption that target variable follows lognormal distribution in 10 bp coverage bands separately for BRCA1/2 and CFTR loci. Datasets with outlying sensitivity for BRCA1/2 variants detection were significantly enriched with ones generated with SureSelect Human All Exon V4 panel (Figure 4A) (observed 81% versus expected 9%, *P*-value 1.2e–11). Coverage uniformity of such datasets did not differ from total sample (*P*-value of 0.21). Notable sensitivity drop which was observed across SureSelect V4 datasets was primarily due to inconsistent coverage of the rs80357783 variant site, comprising 18% of total BRCA1/2 allele count and, thus, accounting for 94% of the total estimated false negative rate in average across such outlying datasets. Coverage of the principal rs80357783 variant was only 4% of mean loci coverage across datasets with outlying sensitivity, and 18% across total sampling. In part, such inconsistent, low coverage could be due to the variant site not being directly covered with hybridization probes, with the nearest probe mapping at a distance of 14 bp, according to the panel specification provided by the manufacturer. For the rest of the datasets with the outlying sensitivity to detect BRCA1/2 variants, approximately 90% of the false negative rate was split among 64 variants, on average. These datasets were characterized by lower coverage uniformity (mean evenness score of 0.61 versus 0.79, *P*-value 0.007). As for CFTR related analysis, 85% of the sensitivity drop cases were associated with the high estimated false negative rate of the rs113993960 variant, accounting for the 73% of the clinically significant CFTR alleles. With no saturation for any panel, these cases were also not associated with lower coverage uniformity or total coverage (*P*-value 0.46 and 0.32).

**Figure 5.**
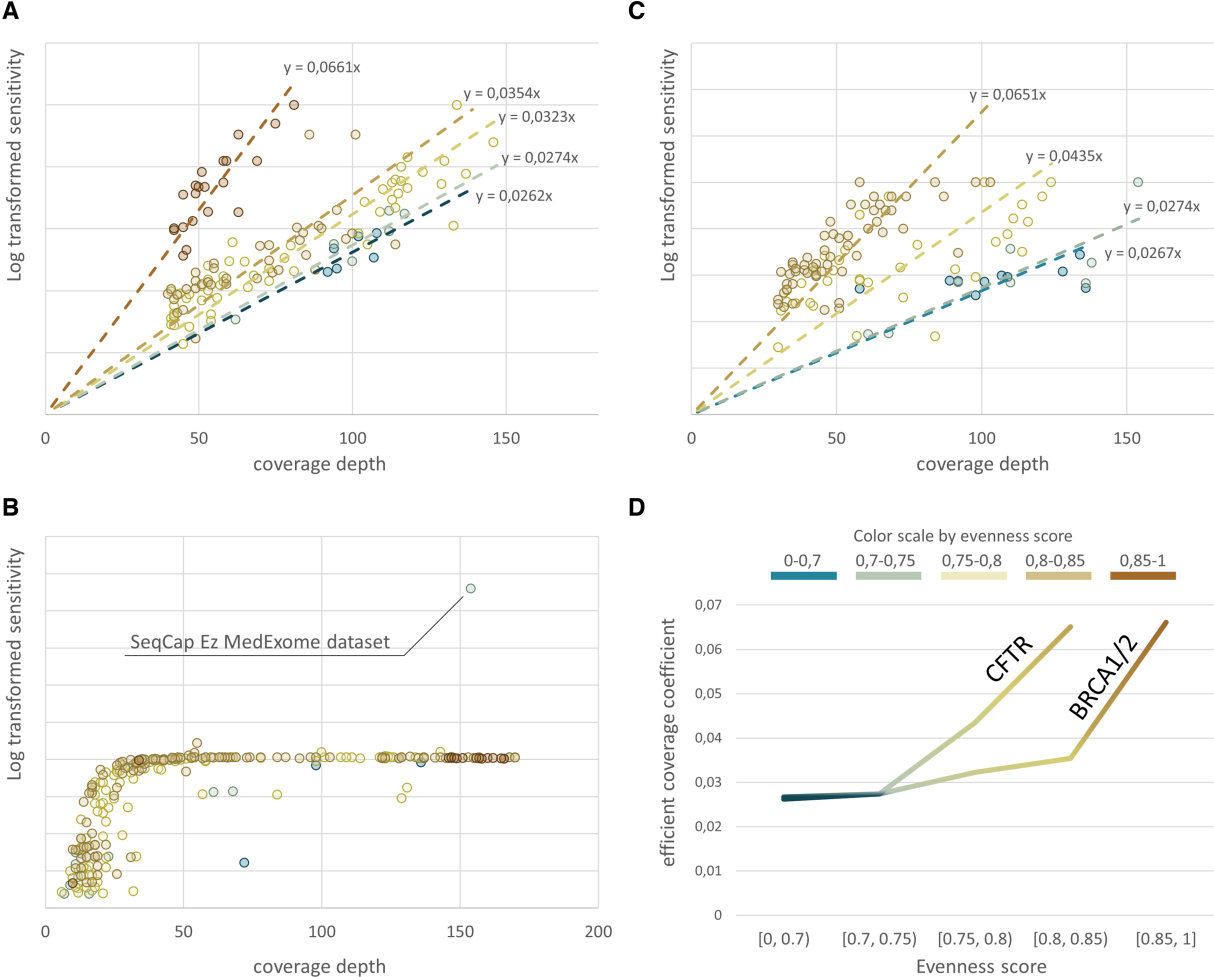
Sensitivity calculation deciphers importance of the coverage uniformity. Dependency of estimated dataset sensitivity on coverage at high coverage depth was assessed employing mapping sensitivity values from [0, 1] to [0, +∞] (see main text for methods). Further intercept free linear regression demonstrates separation of different coverage uniformity ranges by the slope of the line for BRCA1/2 loci (**A**). Log-transformed sensitivity of CFTR variants detection reaches asymptote for the most panels due to sensitivity cap as panels does not cover several intronic CFTR variants (**B**). Excluding these variants from the analysis results in the same separation of different coverage uniformity ranges by the slope of the approximation line (**C**). The rate of growth of the slope as uniformity increases is higher for CFTR loci (**D**).

To decipher the differences of sensitivity calculation at high coverage we performed ${\rm{log}}\left( {\frac{1}{{1 - x}}}\right)$ transformation of the sensitivity to create a map from [0, 1] to [0, +∞]. Further linear regression with no intercept put apart datasets with different evenness score by the slope of the line, demonstrating different benefit gain with an increase in coverage depth for different coverage uniformity. Therefore, the ratio of the slopes for two different evenness score ranges may be interpreted as the ratio between efficient coverage. In this way, BRCA1/2 genes sequencing almost does not benefit with an increase in coverage uniformity up to 0.85, whereas with evenness score of 0.85 and more efficient coverage increases up to almost two times (slope of the line 0.0661 versus 0.0354). It is important to say that datasets with evenness score of 0.85 and higher across BRCA1/2 loci were limited to the ones generated with SeqCap EZ v3.0 panel. These datasets were obtained in course of diverse SRA projects, thus, limiting the interpretation of these results and its application for any other dataset except those analysed here. Nevertheless, linear regression across datasets generated by different panels had not demonstrated significantly different slope in evenness score ranging from [0.7, 0.75) to [0.75, 0.8) (*P*-value ranging from 0.4 to 0.84), for different panels. Overall, these findings demonstrate the utility of evenness score interpretation in terms of the efficient coverage and how coverage uniformity influences dataset quality for calling clinically relevant variants (Figure 5D). Since most panels pertain sensitivity cap as was demonstrated before, efficient coverage of CFTR reaches asymptote as the coverage depth growth, with an exception for SeqCap MedExome Kit which covers commonly mistargeted intronic CFTR variants (Figure 5B). After removing these variants from the reference VCF-file database, linear regression demonstrated the same separation of datasets with varying coverage uniformity by the slope of the lines (Figure 5C), even if their rate of efficient coverage growth was higher as compared to BRCA1/2 (Figure 5D).

### Sensitivity calculation for comparative analysis of different sequencing approaches

**Figure 6.**
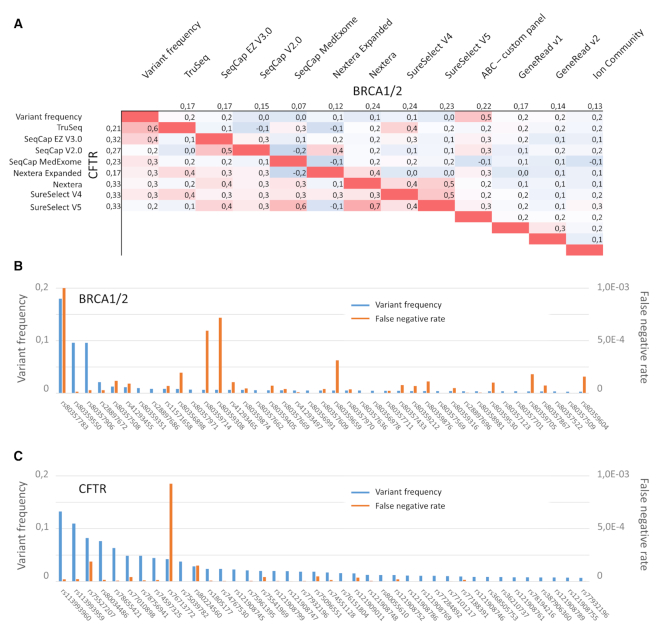
Use of the EphaGen to decipher differences between target panels. During sensitivity calculation false negative rate is estimated for each variant from reference database. As this rate is limited by the prior probability of the variant, one may expect correlation between these two values. Spearman rang correlation was therefore calculated for all panels between their false negative rate as well as allele prior probability (**A**). (B, C) Top 40 variants by frequency and corresponding average false negative rate for BRCA1/2 (**B**) and CFTR (**C**) related analysis.

In addition to the sensitivity calculation EphaGen outputs Single Site Sensitivity for each variant site from a reference database in VCF-file format. Combined with the allele prior probability, it allows calculating estimated false negative rate by the following formula: FN = AF*(1 – Single Site Sensitivity), where AF is prior allele probability, defined by reference variant database. We sought to estimate how calculated this way false negative rate varies among different variant sites for each locus across different panels. For this, we performed downsampling of all datasets to 20 000 and 4000 reads mapping to BRCA1/2 and CFTR loci respectively, as this amount of sequencing data is enough to reach 99% of the sensitivity cap in average for each loci, as was previously shown. Since the false negative rate is limited by the allele prior probability, one may expect a correlation between these two variables. However, spearman rang correlation between average false negative rate across datasets employing the single panel was low, though overall higher for CFTR-related analysis (Figure 6B, C). Moreover, the correlation overall was low between false negative rates produced by different panels (Figure 6A), pointing out that different panels are prone to produce false negatives at different sites. Overall, this indicates that selection of the panel to perform sequence of the selected loci should be performed in accordance with the population of interest, as some populations may harbor founder mutations when some panels but not the others are prone to produce high false negative rate for these particular mutations.

**Figure 7.**
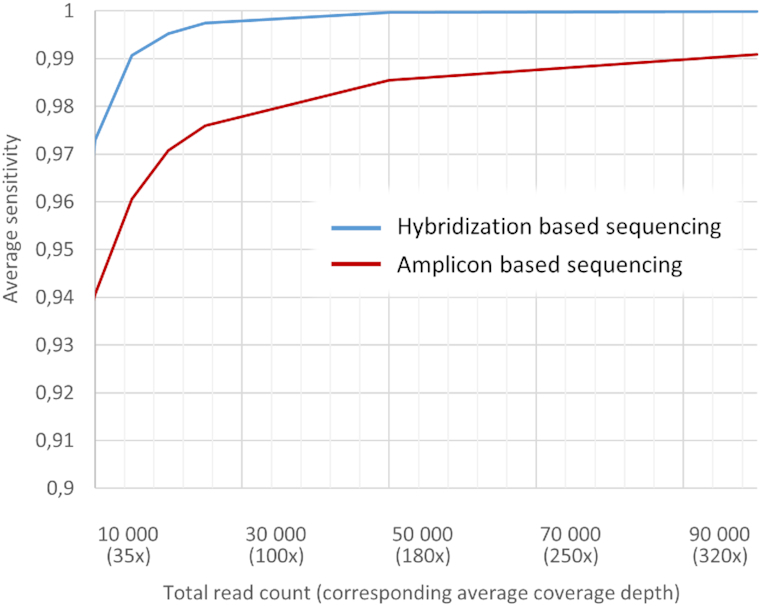
Comparison of the estimated sensitivity for hybridization based and amplicon based sequencing approaches. Downsampling all datasets to the fixed number of reads mapping to the target loci (BRCA1/2) demonstrated overall performance superiority for hybridization based enrichment strategy over amplicon based technology. Sensitivity calculation was performed across all datasets generated employing specific enrichment strategy, excluding outliers.

As amplicon based BRCA1/2 targeted sequencing aims at shorter regions in total, compared to WXS sequencing, this allows performing sequencing at deeper coverage. Sequencing coverage for datasets generated with amplicon-based BRCA1/2 targeted panels was at 4200× in average versus only 140× for WXS panels. Nevertheless, after excluding outliers, estimated sensitivity of these datasets was only slightly higher with 98.4% sensitivity in average (versus 97.5% for WXS) and 25% (versus 35% for WXS) of the datasets reach sensitivity of 99.9% and 7.5% (11.5% for WXS) reach the sensitivity of 100%, considering, that sensitivity estimation is limited by 5°. Off note, since some of the amplicon-based datasets were generated employing semiconductor sequencing (see Materials and Methods for details), this should affect sensitivity calculation as this technology tends to produce data with lower Phred read quality. However, we did not observe any trend towards lower sensitivity of these datasets (mean versus other amplicon-based datasets: 99.9 versus 98.2, *P*-value < 0.0001), which could be explained either by ultra-deep coverage or by the fact that only Ion Ampliseq Custom Design panel was used to generate libraries that were sequenced with semiconductor technology, so bias could be introduced by enrichment strategy. In order to perform the head-to-head comparison of both enrichment strategies, all datasets were downsampled to resemble conditions when a fixed number of reads are generated by sequencing (Figure 7A). As a result, hybridization based enrichment strategy outperformed amplicon-based in all ranges of coverage depth. While understanding that enrichment strategy is selected according to cost-analysis or other prevailing factors, our analysis points to an advantage of hybridization approaches over amplicon based ones.

**Figure 8.**
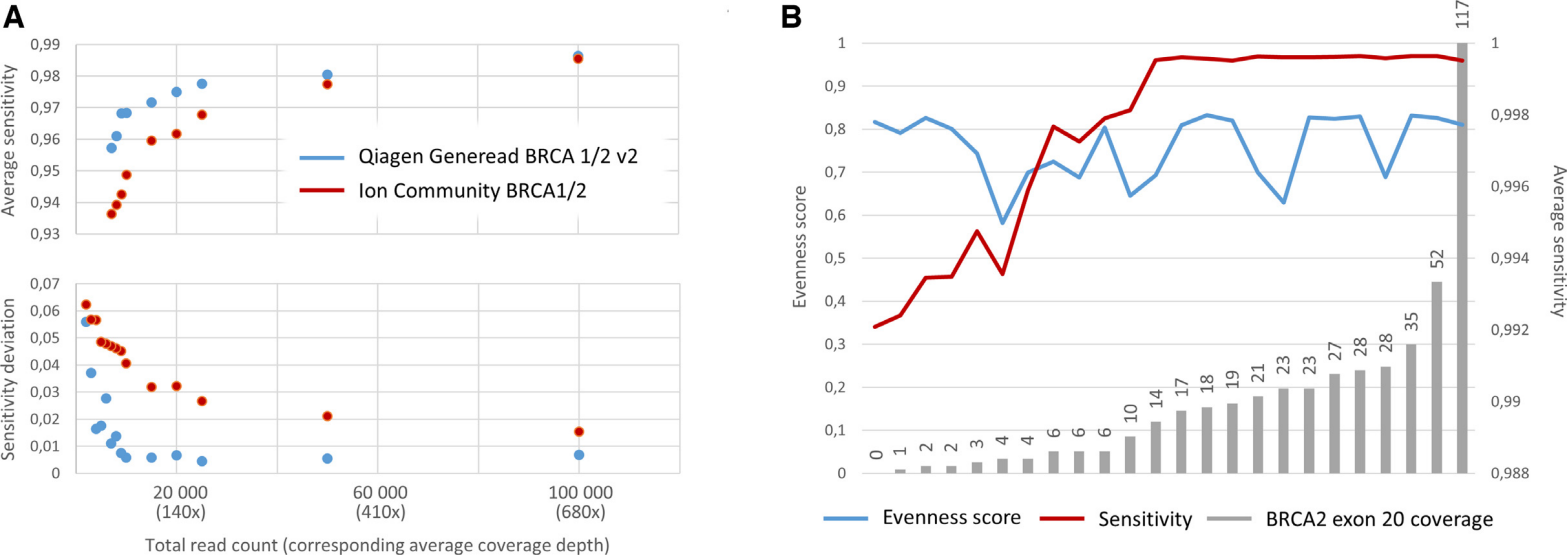
Head-to-head comparison of the Qiagen Generead BRCA 1/2 v2 and Ion Community BRCA1/2 panels. Two panels were characterized by close sensitivity values given the read count as is (mean 99.8% versus 99.7% respectively, *P*-value 0.08). (**A**) Downsampling analysis demonstrated superiority of the Qiagen Generead BRCA 1/2 v2 panel in terms of mean sensitivity and sensitivity standard deviation. (**B**) Datasets generated with Ion Community BRCA1/2 panels in addition to high sensitivity standard deviation were characterized by high coverage uniformity variation (blue line). However, low coverage uniformity was not in correlation with low sensitivity (red line). False negative rate estimation for each variant site illustrated that datasets, generated with Ion Community BRCA1/2 panel, demonstrated that 5 variant sites mapping to BRCA2 exon 20 were characterized with low coverage across datasets with low sensitivity.

Further, we performed head-to-head performance comparison of the amplicon-based panels based on the sensitivity calculation. We observed that two panels, namely Qiagen Generead BRCA 1/2 v2 and Ion Community BRCA1/2 panel were characterized by close sensitivity values given the read count as is (mean 99.8% versus 99.7% respectively, *P*-value 0.08). Downsample analysis demonstrated superiority of the sensitivity value for Generead panel in all coverage depth ranges, though with no statistical significance in any (Figure 8A). What's more important we observed higher sensitivity standard deviation for Ion Community BRCA1/2 panel in coverage depth ranges (Figure 8A). This was correlated to the higher standard deviation in evenness score as several datasets demonstrated drop in coverage uniformity (Figure 3A), though not associated with drop in sensitivity (Spearman correlation coefficient, 0.33, *P*-value 0.11) (Figure 8B). As we studied variants demonstrating low single site sensitivity we identified that sensitivity drop was associated with the undercoverage of the five variant sites located within exon 20 comprising for a total of 0.7% of the BRCA2 gene (Spearman correlation coefficient 0.89, *P*-value < 0.001) (Figure 8B). Of note, BRCA2 exon 20 skipping was not reported in any sample in the original study (13). Limiting the analysis to datasets with descent BRCA2 exon coverage (15× and more) demonstrated contrariwise inferiority of the Generead panel compared to the Ion Community BRCA1/2 panel (mean sensitivity 99.95% versus 99.8%, *P*-value < 0.0001) despite the higher estimated sensitivity cap (Figure 3A). This points, that though datasets generated by Ion Community BRCA1/2 panel were characterized by both high standard deviation of the evenness score and standard deviation of the sensitivity, several datasets with low coverage uniformity still demonstrated high sensitivity due to ultra-deep coverage. Meanwhile, sensitivity analysis allowed identifying the low quality cases, and illustrating the applicability of the sensitivity calculation as the quality control metric in routine practice.

Furthermore, after excluding outliers, two panels, namely Qiagen Generead BRCA 1/2 v1 and Ampliseq Custom Design panel were characterized by close coverage uniformity (average evenness score of 0.76 versus 0.76, *P*-value 0.056). However, the last was characterized by the superior sensitivity estimates (mean 99.9% versus 97.8%) which is correlated with the sensitivity cap estimations (Figure 3A), exposing the utility of the sensitivity calculation for comparative analysis.

### Sensitivity calculation for quality control in routine practice

**Figure 9.**
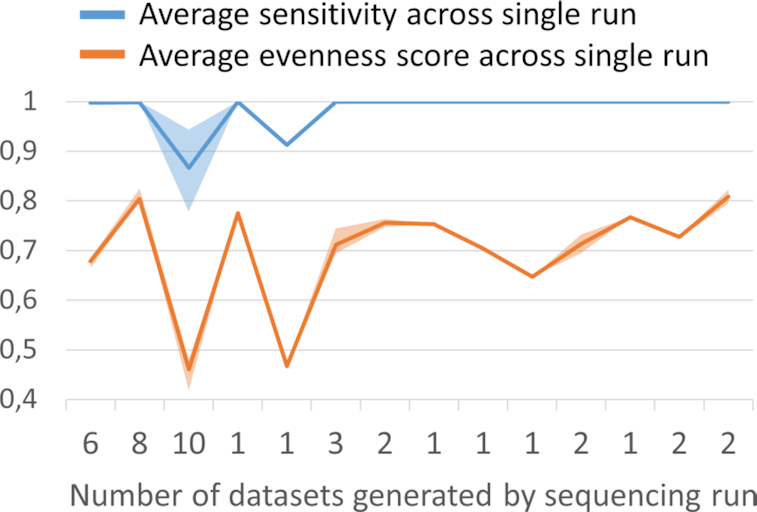
Use of the sensitivity estimation in routine testing. Average sensitivity and coverage uniformity variation across 14 different BRCA1/2 sequencing runs of the total of 43 sequencing libraries prepared employing Ampliseq Custom Design panel demonstrates the utility of sensitivity calculation as quality control metric. Shaded fill shows 75% quartile.

As datasets sequenced employing Ion Ampliseq Custom Design panel were generated through the number of sequencing runs, we retrospectively assessed how sensitivity calculation can be used for quality control in routine practice. With the total of 43 datasets generated through 14 separate runs, average sensitivity within a single run was over 99.9% in 78% cases, while across two runs (14%) we observed average sensitivity drop down to 86% and 91% (Figure 9). Average sensitivity drop within single run was associated with coverage uniformity drop (Spearman correlation coefficient 0.75, *P*-value < 0.001). We identified that these cases were also associated with single primer pool under-representation (average coverage ratio 184 versus 1.3 in other cases). Drawing parallels with the series of datasets generated employing Ion Community BRCA1/2 panel, where coverage uniformity drop was not associated with the sensitivity drop (Figure 8B), this finding highlights the utility of sensitivity calculation for routine quality control.

## DISCUSSION

Progress in the development of appropriate performance measures is essential to advancing applied science and engineering. Such measures not only allow manufactures to control the quality of the scalable process, but also to perform reasonable comparison between current solutions to adopt the best one. From this perspective, control metrics providing a single arbiter number are beneficial as they allow ubiquitous head-to-head comparison of complex systems with numerous variables (14).

As NGS moves forward into routine medical genetics experiment, its clinical implementation requires higher quality standards. Several recently developed bioinformatics control metrics (15–18), do not go further than convenient measures of the percentage of aligned reads, percentage of unique reads, percentage of bases corresponding to targeted sequences, uniformity of coverage, density of clusters, and percentage of targeted bases with no coverage (19). These metrics treat all genome positions equally. Notably, disease-causing mutations tend to be found at certain positions, in particular due to founder effect (20–24), and also to functional constraints. Considering that novel variants are tough for confident classification as pathogenic (11,25,26) and, in absence of segregation data, may require additional laboratory research (11), while variants of uncertain significance (VUS) not always can be directly translated into clinical recommendations (27–30). Without de-emphasizing detecting VUS and novel variants in routine laboratory practice, there is a need for shifting the focus of quality control step to genome positions carrying recurring, rather than random variants.

Here, we describe EphaGen, a novel approach to performance measurement in routine clinical NGS testing. Given a single NGS dataset in BAM format and pre-compiled VCF-file of reference clinically relevant variants with known relative allele prior probability (i.e. allele count in affected population) it associates this dataset with a single parameter, resembling diagnostic sensitivity.

As we demonstrate in this study, most valuable bioinformatics metrics, namely coverage depth and coverage uniformity, are rid with interpretation problems. This way diverse average coverage depth may possess varying efficiency (Figure 4) as the clinical application varies, coverage uniformity is not ubiquitous across diverse loci (Figure 3C) and may reflect the diverse efficiency of coverage for different variant spectrum (Figure 5). Meanwhile, sensitivity calculation is genuinely interpretable in clinical terms and present ubiquitous performance measure for a single application defined by reference VCF-file of clinically relevant variants. For reference database with uniformly disturbed allele prior probabilities, calculated sensitivity resembles convenient coverage uniformity metrics. However, the higher dispersion allele prior probability distribution pertains, the more sequencing datasets may be observed with decent coverage uniformity, but not sensitivity as in some cases it may significantly drop. Using BRCA1/2 and CFTR screenings applications as relevant examples, we have performed the extensive study on how sensitivity calculation may be used as quality control metrics (Figures 8B and 9) as well as the analysis tool for a head-to-head comparison between different sequencing approaches (Figures 6A, 7 and 8A). The data used for the analysis was highly heterogeneous due to the variability of the applied library preparation methods, study designs used to generate test datasets etc. Therefore, these results cannot be considered as doubtless conclusion on the comparative performance of the studied enrichment strategies in the application of BRCA1/2 or CFTR screening. Nevertheless, it clearly demonstrates a high value of sensitivity calculation as the performance measure and its advantages in specified applications over coverage depth or coverage uniformity measurements.

However, the described approach possesses several inherent limitations. First of all, EphaGen metrics are aimed only at single nucleotide variants or small insertions/deletions (generally up to 50 bp.). For now, genome copy number variations within a single gene, like single or several exon deletions or insertions, manifest higher importance in medical genetics as sequencing techniques are advanced and novel variants of this type are identified and their prevalence is refined (31–34). Meanwhile, large gene rearrangements are not only disregarded by the described method but also if occurred in homozygote may serve as a source of misleading sensitivity estimations as falsely treated as coverage artifacts.

Furthermore, as sensitivity calculation is based on the relative allele frequency data, it may possess population bias as diverse ancestral groups demonstrate a variable spectrum of founder mutations (25,35,36). Finally, allele prior probability overestimation of the frequent founder mutations may be introduced in the allele counts as these variants are of particular interest for different research groups. Thus, this may produce bias in false negative rate estimations towards higher rate for variants with high allele count.

In conclusion, we have described a novel approach to performance measurement in routine clinical NGS testing. Given the spectrum of clinically relevant variants, it associates single NGS datasets with a single number, resembling diagnostic sensitivity. On the examples of BRCA1/2 and CFTR screenings applications, we have performed an extensive study on how sensitivity estimation using described approach may be used as quality control metrics as well as the analysis tool for a head-to-head comparison between different sequencing approaches. As developed approach provides single arbiter number, it can be easily implemented into existing clinical workflows as a measure of quality control compatible with Westgard rules in a manner essentially similar to that for mean coverage depth. EphaGen-provided sensitivity estimates may be implemented after the variant calling stage. For example, when only limited amount of pathogenic variants is expected, for instance, only one pathogenic variant for germline BRCA1 analysis, or two variants for CFTR analysis, and variant calling algorithm has failed to identify it, EphaGen may be used as a referee evaluating quality of the data and guiding possible re-analysis.

## DATA AVAILABILITY

EphaGen source code available at https://github.com/m4merg/EphaGen. EphaGen Docker image available at https://hub.docker.com/r/m4merg/ephagen. DNA sequencing data have been deposited with the Sequence Read Archive under accession number SRP173561.

## References

[1] Wilfert A.B., Sulovari A., Turner T.N., Coe B.P., Eichler E.E. Recurrent de novo mutations in neurodevelopmental disorders: properties and clinical implications. Genome Med. 2017; 9:101. 29179772 10.1186/s13073-017-0498-x PMC5704398

[2] Matthijs G., Souche E., Alders M., Corveleyn A., Eck S., Feenstra I., Race V., Sistermans E., Sturm M., Weiss M. et al. Guidelines for diagnostic next-generation sequencing. Eur. J. Hum. Genet. 2016; 24:2–5. 26508566 10.1038/ejhg.2015.226 PMC4795226

[3] Pagin A., Devos A., Figeac M., Truant M., Willoquaux C., Broly F., Lalau G. Applicability and efficiency of NGS in routine diagnosis: in-depth performance analysis of a complete workflow for cftr mutation analysis. PLoS One. 2016; 11:e0149426. 26900683 10.1371/journal.pone.0149426 PMC4762772

[4] Park H.S., Park S.J., Kim J.Y., Kim S., Ryu J., Sohn J., Park S., Kim G.M., Hwang I.S., Choi J.R. et al. Next-generation sequencing of BRCA1/2 in breast cancer patients: potential effects on clinical decision-making using rapid, high-accuracy genetic results. Ann. Surg. Treat. Res. 2017; 92:331–339. 28480178 10.4174/astr.2017.92.5.331 PMC5416916

[5] Jamuar S.S., Tan E.C. Clinical application of next-generation sequencing for Mendelian diseases. Hum. Genomics. 2015; 9:10. 26076878 10.1186/s40246-015-0031-5 PMC4482154

[6] Szabo C., Masiello A., Ryan J.F., Brody L.C. The breast cancer information core: database design, structure, and scope. Hum. Mutat. 2000; 16:123–131. 10923033 10.1002/1098-1004(200008)16:2<123::AID-HUMU4>3.0.CO;2-Y

[7] Li H., Durbin R. Fast and accurate short read alignment with Burrows-Wheeler transform. Bioinformatics. 2009; 25:1754–1760. 19451168 10.1093/bioinformatics/btp324 PMC2705234

[8] Richards S., Aziz N., Bale S., Bick D., Das S., Gastier-Foster J., Grody W.W., Hegde M., Lyon E., Spector E. et al. Standards and guidelines for the interpretation of sequence variants: a joint consensus recommendation of the American college of medical genetics and genomics and the association for molecular pathology. Genet. Med. 2015; 17:405–424. 25741868 10.1038/gim.2015.30 PMC4544753

[9] Li H., Ruan J., Durbin R. Mapping short DNA sequencing reads and calling variants using mapping quality scores. Genome Res. 2008; 18:1851–1858. 18714091 10.1101/gr.078212.108 PMC2577856

[10] Link W.A. Nonidentifiability of population size from capture-recapture data with heterogeneous detection probabilities. Biometrics. 2003; 59:1123–1130. 14969493 10.1111/j.0006-341x.2003.00129.x

[11] Castellani C. CFTR2 team CFTR2: how will it help care. Paediatr. Respir. Rev. 2013; 14:2–5. 10.1016/j.prrv.2013.01.006 23466340

[12] Oexle K. Evaluation of the evenness score in next-generation sequencing. J. Hum. Genet. 2016; 61:627–632. 27074764 10.1038/jhg.2016.21

[13] Ellison G., Huang S., Carr H., Wallace A., Ahdesmaki M., Bhaskar S., Mills J. A reliable method for the detection of BRCA1 and BRCA2 mutations in fixed tumour tissue utilising multiplex PCR-based targeted next generation sequencing. BMC Clin. Pathol. 2015; 15:5. 25859162 10.1186/s12907-015-0004-6 PMC4391122

[14] Horton P. A commentary on evaluation of the evenness score in next-generation sequencing. J. Hum. Genet. 2016; 61:575. 27074765 10.1038/jhg.2016.29 PMC4960519

[15] Tawari N.R., Seow J.J.W., Perumal D., Ow J.L., Ang S., Devasia A.G., Ng P.C. ChronQC: a quality control monitoring system for clinical next generation sequencing. Bioinformatics. 2018; 34:1799–1800. 29300845 10.1093/bioinformatics/btx843

[16] Lo C.C., Chain P.S. Rapid evaluation and quality control of next generation sequencing data with FaQCs. BMC Bioinformatics. 2014; 15:366. 25408143 10.1186/s12859-014-0366-2 PMC4246454

[17] Zhou Q., Su X., Wang A., Xu J., Ning K. QC-Chain: fast and holistic quality control method for next-generation sequencing data. PLoS One. 2013; 8:e60234. 23565205 10.1371/journal.pone.0060234 PMC3615005

[18] Doig K.D., Fellowes A., Bell A.H., Seleznev A., Ma D., Ellul J., Li J., Doyle M.A., Thompson E.R., Kumar A. et al. PathOS: a decision support system for reporting high throughput sequencing of cancers in clinical diagnostic laboratories. Genome Med. 2017; 9:38. 28438193 10.1186/s13073-017-0427-z PMC5404673

[19] Endrullat C., Glökler J., Franke P., Frohme M. Standardization and quality management in next-generation sequencing. Appl. Transl. Genom. 2016; 10:2–9. 27668169 10.1016/j.atg.2016.06.001 PMC5025460

[20] Ossa C.A., Torres D. Founder and recurrent mutations in BRCA1 and BRCA2 genes in latin American countries: state of the art and literature review. Oncologist. 2016; 21:832–839. 27286788 10.1634/theoncologist.2015-0416 PMC4943386

[21] Olsson K.S., Wålinder O., Jansson U., Wilbe M., Bondeson M.L., Stattin E.L., Raha-Chowdhury R., Williams R. Common founder effects of hereditary hemochromatosis, Wilson′s disease, the long QT syndrome and autosomal recessive deafness caused by two novel mutations in the WHRN and TMC1 genes. Hereditas. 2017; 154:16. 29270100 10.1186/s41065-017-0052-2 PMC5735936

[22] Hishinuma A., Fukata S., Nishiyama S., Nishi Y., Oh-Ishi M., Murata Y., Ohyama Y., Matsuura N., Kasai K., Harada S. et al. Haplotype analysis reveals founder effects of thyroglobulin gene mutations C1058R and C1977S in Japan. J. Clin. Endocrinol. Metab. 2006; 91:3100–3104. 16720658 10.1210/jc.2005-2702

[23] Evans J.A. Old meets new: identifying founder mutations in genetic disease. CMAJ. 2015; 187:93–94. 25602001 10.1503/cmaj.141509 PMC4312142

[24] Chiu Y.H., Chang Y.C., Chang Y.H., Niu D.M., Yang Y.L., Ye J., Jiang J., Okano Y., Lee D.H., Pangkanon S. et al. Mutation spectrum of and founder effects affecting the PTS gene in East Asian populations. J. Hum. Genet. 2012; 57:145–152. 22237589 10.1038/jhg.2011.146

[25] Santonocito C., Scapaticci M., Guarino D., Bartolini A., Minucci A., Concolino P., Scambia G., Paris I., Capoluongo E. Identification of twenty-nine novel germline unclassified variants of BRCA1 and BRCA2 genes in 1400 Italian individuals. Breast. 2017; 36:74–78. 29020660 10.1016/j.breast.2017.09.007

[26] Yang C., Jairam S., Amoroso K.A., Robson M.E., Walsh M.F., Zhang L. Characterization of a novel germline BRCA1 splice variant, c.5332+4delA. Breast Cancer Res. Treat. 2018; 168:543–550. 29185120 10.1007/s10549-017-4595-8 PMC6788766

[27] Vears D.F., Niemiec E., Howard H.C., Borry P. Analysis of VUS reporting, variant reinterpretation and recontact policies in clinical genomic sequencing consent forms. Eur. J. Hum. Genet. 2018; 26:1743–1751. 30143804 10.1038/s41431-018-0239-7 PMC6244391

[28] Balmaña J., Digiovanni L., Gaddam P., Walsh M.F., Joseph V., Stadler Z.K., Nathanson K.L., Garber J.E., Couch F.J., Offit K. et al. Conflicting interpretation of genetic variants and cancer risk by commercial laboratories as assessed by the prospective registry of multiplex testing. J. Clin. Oncol. 2016; 34:4071–4078. 27621404 10.1200/JCO.2016.68.4316 PMC5562435

[29] Vail P.J., Morris B., van Kan A., Burdett B.C., Moyes K., Theisen A., Kerr I.D., Wenstrup R.J., Eggington J.M. Comparison of locus-specific databases for BRCA1 and BRCA2 variants reveals disparity in variant classification within and among databases. J. Commun. Genet. 2015; 6:351–359. 10.1007/s12687-015-0220-x PMC4567983 25782689

[30] Lincoln S.E., Yang S., Cline M.S., Kobayashi Y., Zhang C., Topper S., Haussler D., Paten B., Nussbaum R.L. Consistency of BRCA1 and BRCA2 variant classifications among clinical diagnostic laboratories. JCO Precis. Oncol. 2017; 1:doi:10.1200/PO.16.00020. 10.1200/PO.16.00020 PMC5542009 28782058

[31] Lefterova M.I., Shen P., Odegaard J.I., Fung E., Chiang T., Peng G., Davis R.W., Wang W., Kharrazi M., Schrijver I. et al. Next-generation molecular testing of newborn dried blood spots for cystic fibrosis. J. Mol. Diagn. 2016; 18:267–282. 26847993 10.1016/j.jmoldx.2015.11.005 PMC4816703

[32] Judkins T., Rosenthal E., Arnell C., Burbidge L.A., Geary W., Barrus T., Schoenberger J., Trost J., Wenstrup R.J., Roa B.B. Clinical significance of large rearrangements in BRCA1 and BRCA2. Cancer. 2012; 118:5210–5216. 22544547 10.1002/cncr.27556 PMC3532625

[33] Palanca S., de Juan I., Perez-Simó G., Barragán E., Chirivella I., Martínez E., Fuster O., Bolufer P. The deletion of exons 3–5 of BRCA1 is the first founder rearrangement identified in breast and/or ovarian cancer Spanish families. Fam. Cancer. 2013; 12:119–123. 23117300 10.1007/s10689-012-9579-6

[34] Schmidt A.Y., Hansen T.V.O., Ahlborn L.B., Jønson L., Yde C.W., Nielsen F.C. Next-generation sequencing-based detection of germline copy number variations in BRCA1/BRCA2: validation of a one-step diagnostic workflow. J. Mol. Diagn. 2017; 19:809–816. 28822785 10.1016/j.jmoldx.2017.07.003

[35] Hall M.J., Reid J.E., Burbidge L.A., Pruss D., Deffenbaugh A.M., Frye C., Wenstrup R.J., Ward B.E., Scholl T.A., Noll W.W. BRCA1 and BRCA2 mutations in women of different ethnicities undergoing testing for hereditary breast-ovarian cancer. Cancer. 2009; 115:2222–2233. 19241424 10.1002/cncr.24200 PMC2771545

[36] Palmero E.I., Carraro D.M., Alemar B., Moreira M.A.M., Ribeiro-Dos-Santos Â., Abe-Sandes K., Galvão H.C.R., Reis R.M., de Pádua Souza C., Campacci N. et al. The germline mutational landscape of BRCA1 and BRCA2 in Brazil. Sci. Rep. 2018; 8:9188. 29907814 10.1038/s41598-018-27315-2 PMC6003960

